# Regulatory T cells isolated from endometriotic peritoneal fluid express a different number of Toll-like receptors

**DOI:** 10.31744/einstein_journal/2020AO5294

**Published:** 2020-03-19

**Authors:** Camila Hernandes, Bárbara Yasmin Gueuvoghlanian-Silva, Vitor Ulisses Monnaka, Natalia Mazini Ribeiro, Welbert de Oliveira Pereira, Sérgio Podgaec

**Affiliations:** 1 Hospital Israelita Albert Einstein São PauloSP Brazil Hospital Israelita Albert Einstein, São Paulo, SP, Brazil.; 2 Instituto Israelita de Ensino e Pesquisa Albert Einstein Hospital Israelita Albert Einstein São PauloSP Brazil Instituto Israelita de Ensino e Pesquisa Albert Einstein, Hospital Israelita Albert Einstein, São Paulo, SP, Brazil.; 3 Faculdade Israelita de Ciências da Saúde Albert Einstein Hospital Israelita Albert Einstein São PauloSP Brazil Faculdade Israelita de Ciências da Saúde Albert Einstein, Hospital Israelita Albert Einstein, São Paulo, SP, Brazil.

**Keywords:** T-lymphocytes, regulatory, Toll-like receptors, Immune system, Endometriosis

## Abstract

**Objective:**

To analyze and compare the expression of Toll-like receptors by regulatory T cells present in the peritoneal fluid of patients with and without endometriosis.

**Methods:**

Regulatory T cells were isolated from peritoneal fluid of women with and without endometriosis, collected during surgery, and mRNA was extracted for analysis of Toll-like receptors expression by reverse-transcriptase polymerase chain reaction.

**Results:**

Patients with endometriosis presented regulatory T cells expressing a larger number and variety of Toll-like receptors when compared to regulatory T cells from patients in the Control Group. Toll-like receptor-1 and Toll-like receptor-2 in regulatory T cells were expressed in both groups. All other expressed Toll-like receptors types were only found in regulatory T cells from the Endometriosis Group.

**Conclusion:**

Patients with endometriosis had peritoneal regulatory T cells expressing various Toll-like receptors types.

## INTRODUCTION

The pathogenesis of endometriosis remains unclear.^1,2^ The most accepted theory is the one advanced by Sampson;^3^ it proposes that during menses, the endometrial cells are transported through the fallopian tubes by reverse flow to the peritoneal cavity, where they attach to the serosal surfaces. However, although retrograde menstruation occurs in 90% of women, only approximately 10% develop endometriosis.^4,5^ In view of this discrepancy, complementary theories suggest that the immune response of endometriosis patients is altered, and it fails to eliminate the ectopic endometrial implants.^6-10^

As an example, regulatory T cells (Treg) are a subpopulation of specialized immune regulation T lymphocytes, which have been already related to the development of endometriosis.^9^

Although Treg are associated with the production of anti-inflammatory cytokines, there is evidence that under inflammatory conditions, they can also express inflammatory cytokines and, when unregulated, may act to maintain the disease.^11^

Recently, a novel theory was put forward, associating the presence of microorganisms to development of endometriosis.^11,12^ It originated from studies indicating that there was more bacterial contamination in menstrual blood, mainly by *Escherichia coli*, and higher endotoxin levels in the peritoneal fluid of women with endometriosis, as compared to patients without the disease.^11,13^ Another study showed that, in women with endometriosis, the presence of *Mycoplasma genitalium* is associated with down-regulation of genes associated with inflammatory response.^14^ The idea is that during retrograde menstruation, endometrial tissue and microorganisms are carried to the peritoneal cavity,^12^ with subsequent attachment, proliferation, differentiation and invasion by these cells.^2^

Some studies have suggested microorganisms can be directly detected by Treg through Toll-like receptors (TLRs), which can modulate the suppressive functions of Tregs, and the suppressive function of Treg cells could be enhanced or reversed by TLR.^15^

Considering that women with endometriosis would indeed have more microorganisms in the peritoneal cavity that healthy women, and that microorganisms express and secrete TLR agonists, which could affect Treg cells, we raise the hypothesis there is a difference between TLR expression in Treg cells isolated from patients with endometriosis when compared with controls.

## OBJECTIVE

To analyze and compare the expression of Toll-like receptors by regulatory T cells isolated from peritoneal fluid obtained from patients with and without endometriosis.

## METHODS

### Study population

This case-control study was conducted at *Hospital Israelita Albert Einstein* (HIAE), São Paulo (SP), Brazil, from January 1^st^ to December 1^st^, 2018. The study was approved by the HIAE Research Ethics Committee, number 563.939, CAAE: 25915014.2.0000.0071, and all patients signed an Informed Consent Form. Patients were divided into two groups based on surgical findings: with and without endometriosis. The diagnosis of endometriosis depended on finding the characteristic lesions during surgery and subsequent confirmation by histological analysis. Control Group consisted of women with no evidence of endometriosis and who had been operated due to benign ovarian cysts, uterine fibroids, diagnostic laparoscopy or tubal ligation.

A total of 29 patients were included in this study: 23 with endometriosis and 6 controls. Inclusion criteria were women aged 18 to 40 years with eumenorrheic cycles, who underwent laparoscopic surgery (to confirm or rule out endometriosis). The exclusion criteria were women with autoimmune, inflammatory, and/or neoplastic disease and/or taking hormone therapy, including gonadotropin-releasing hormone (GnRH) analogues, progestins, or combined contraceptives, for 3 months before surgery.

For sample collection, just after the insertion of the auxiliary trocar in laparoscopic surgery, all peritoneal fluid deposited in the anterior and/or posterior *cul-de-sac* was collected with a laparoscopic needle.

Patients were clinically characterized by the presence of deep dyspareunia, dysmenorrhea, chronic pelvic pain, abortions, infertility, menstrual cycle phase, age, race and body mass index (BMI). Dysmenorrhea was measured using the Visual Analog Scale (VAS), a validated score in which a 10cm line represents a grade between “no pain” and “worst paint”.

### Isolation and characterization of regulatory T cells from peritoneal fluid

Peritoneal fluid was processed to isolate lymphocytes. Erythrocytes were lysed with lysis buffer at room temperature for 15 minutes. Then cells were washed with phosphate buffered saline (PBS), counted and frozen in fetal bovine serum (FBS) (Gibco, USA) with 10% dimethyl sulfoxide (DMSO) (Sigma-Aldrich, USA) and cryopreserved in liquid nitrogen until use, when they were thawed under agitation, in water bath at 37°C. The cells were washed by centrifugation (250g, 8 minutes, 8°C) initially with 0.01M PBS, pH 7.2, and followed by a second washing in culture medium (R-10) (Invitrogen-Gibco, Gaithersburg, MD, USA). Next, the cells were suspended in R-10 medium for counting and evaluation of cell viability in a Countess™ automated cell counter. The CD4^+^CD25^+^CD127^dim^ regulatory T Cell Isolation Kit II (MACS, MiltenyiBiotec, Auburn, CA, USA) was used to separate the cells according to the manufacturer’s instructions. Briefly, non-CD4^+^ and CD127^high^ cells were labelled with a cocktail of biotin-conjugated antibodies (CD8, CD19, CD123 and CD127) and submitted to magnetic separation. This enriched fraction of CD4^+^ T cells was then labelled with anti-CD25 antibody and again underwent magnetic separation. Treg cells (CD4^+^CD25^+^CD127^dim^) were eluted as the positively selected cell fraction. The samples were centrifuged, suspended in 200μLTrizol (TRIzol^®^ reagent, Invitrogen), and frozen at -80°C for extraction of total ribonucleic acid (RNA).

### RNA extraction and messenger RNA expression assay

Total RNA from isolated Tregs was extracted according to a standard Trizol RNA isolation protocol (Invitrogen) and purified (Rneasy Micro Kit™; Qiagen, Dusseldorf, Germany). Ribonucleic acid concentration was determined using a NanoDrop^®^ ND-1000 spectrophotometer (Nano-Drop Technologies, Wilmington, Australia). All samples were stored at -80°C.

Complementary deoxyribonucleic acid (cDNA) synthesis was performed by reverse transcription using the specific ImProm-II™ Reverse Transcription System kit (Promega, Madison, WI, USA). The samples were incubated with the specified volume of Random Primer at 70°C for 5 minutes, and then placed on ice for 5 minutes. Next, the samples were briefly centrifuged and left on ice until the completion of the next step. A reagent mixture containing deoxynucleotide triphosphate (dNTP), MgCl_2_, ImProm-II Reverse Transcriptase (Promega), and ImProm-II™ 5x Reaction Buffer (Promega) was prepared, added to the RNA with Random Primer, and incubated at 25°C for 5 minutes, 42°C for 60 minutes, followed by 10 minutes at 70°C. The resulting cDNAs were stored at -20°C. All reactions were performed at an RNA concentration of 20ng/mL.

Complementary deoxyribonucleic acid pre-amplification was performed using a reagent mix consisting of TaqMan Gene Expression Assays (Applied Biosystems, Foster City, CA, USA) and TE buffer to complete the volume. The tubes with the reagent mix, cDNA samples, and Master Mix Pre-Amp (Applied Biosystems) were incubated at 95°C for 10 minutes, and then for ten cycles of 95°C for 15 minutes, followed by 4 minutes at 60°C. All samples were stored at -20°C.

Messenger RNA expression was determined by reverse-transcriptase polymerase chain reaction (RT-PCR) on an ABI 7500 sequence detection system (Applied Biosystems). Polymerase chain reaction was performed in separate tubes for each reaction and each sample was run in duplicate. TaqMan Gene Expression Assays (Applied Biosystems) were used for target genes TLR1 (Hs00413978_m1), TLR2 (Hs00610101_m1), TLR3 (Hs01551078_m1), TLR4 (Hs00152939_m1), TLR5 (Hs00152825_m1), TLR6 (Hs00271977_s1), TLR7 (Hs00152971_m1), TLR8 (Hs00152972_m1) and TLR9 (Hs00152973_m1).

### Statistical analysis

Data analyses were performed using the R statistical program. A significance level of 0.05 was adopted for all comparisons.

Data distribution and variations of all continuous variables were assessed by Shapiro-Wilk and F tests, respectively. Bearing in mind these analyses and the small sample size, non-parametric tests were used. In order to evaluate clinical differences between the groups that could influence the results, we compared the presence of deep dyspareunia, dysmenorrhea, chronic pelvic pain, abortions, infertility, menstrual cycle phase, age, race and BMI. Abortions, deep dyspareunia and chronic pelvic pain were compared by Fisher’s exact test, considering the small sample size and the 2x2 table. Race, menstrual phase and infertility were compared by χ^2^ test. Age, BMI and dysmenorrhea were compared by Wilcoxon rank-sum test. Since there were missing data on race of four patients and BMI of five patients, the analysis was performed with the available data.

The expression of TLR from Treg cells from peritoneal fluid of Endometriosis and Control Groups was qualitatively analyzed considering the cycle threshold (CT) value of 32 for the categorization of receptor expression in an either “expressed” or “non-expressed” binary qualitative variable.

Subsequently, we compared the specific TLR expression in each group. We also evaluated the TLR expression according to the type of endometriosis. In both cases, Fisher’s exact test was used. The number of TLRs expressed in each group was compared by Wilcoxon rank-sum test. Considering the lower efficiency of this method for small samples, we have also included the Student’s *t* test. The correlation between the number of TLRs expressed and the age of patients with endometriosis was analyzed by Spearman’s test.

## RESULTS

### Clinical parameters

The clinical characteristics are presented in table 1. Infertility was reported by 14 patients in the Endometriosis Group and 2 in the control subjects (p=0.019). The mean of dysmenorrhea at VAS was 8.37 in Endometriosis Group and 5.83 in the control subjects (p=0.027). The other clinical parameters showed no difference between groups.

**Table 1:** Baseline characteristics of endometriosis and control patients

Parameters	Control	Endometriosis	p value
Patients	6	23	
Age	34.7	34.5	0.765
Menstrual phase			0.200
Menstrual	0	2	
Proliferative	4	5	
Secretory	1	9	
Unknown	1	7	
Dysmenorrhea	5.83	8.39	0.027
Chronic pelvic pain	2	15	0.198
Deep dyspareunia	2	14	0.364
Infertility			0.019
Yes	2	14	
No	4	3	
Unknown	0	6	
Abortions	1	4	1

Results expressed as n or mean.

Dysmenorrhea: mean at Visual Analogue Scale. Abortions, deep dyspareunia and chronic pelvic pain were compared by Fisher’s exact test, menstrual phase and infertility by χ^2^ test, age and dysmenorrhea by Wilcoxon rank-sum test.

### Toll-like receptor expression

The expression of TLR in Treg cells obtained from the peritoneal fluid is summarized in table 2. In the Control Group, three out of the six women did not express any TLR in peritoneal Tregs, one expressed only TLR1 and two women expressed only TLR2. Expression of TLR 3 to 9 by peritoneal Tregs was negative in all control subjects.

**Table 2:** Expression of Toll-like receptors by peritoneal regulatory T-cells from patients with endometriosis and controls

TLR	Endometriosis	Control	p value
TLR	12/23 (52.2)	3/6 (50.0)	1.000
TLR1	8/23 (34.8)	1/6 (16.7)	0.633
TLR2	12/23 (52.2)	2/6 (33.3)	0.651
TLR3	1/23 (4.3)	0/6 (0.0)	1.000
TLR4	5/23 (21.7)	0/6 (0.0)	0.553
TLR5	6/23 (26.1)	0/6 (0.0)	0.295
TLR6	2/23 (8.7)	0/6 (0.0)	1.000
TLR7	5/23 (21.7)	0/6 (0.0)	0.553
TLR8	3/23 (13.0)	0/6 (0.0)	1.000
TLR9	0/23 (0.0)	0/6 (0.0)	-

Results expressed by the % of women within the group with and without endometriosis whose Treg cells showed expression of a certain type of TLR.

Expression of a specific Toll-like receptor in the peritoneal regulatory T cells population was considered positive (+; binary qualitative variable) considering the cycle threshold value of 32. Toll-like receptors compared by Fisher’s exact test. TLR: Toll-like receptors.

In the Endometriosis Group, seven patients had peritoneal Tregs expressing three or more TLR, with TLR5 being the most frequent (in six out of seven patients). Four patients expressed only TLR2 and one patient expressed both TLR1 and TLR2, whereas in 11 patients peritoneal Tregs did not express any TLR.

Except for TLR9, all TLRs were found to be expressed (alone or co-expressed) in Tregs from endometriosis patients.

No association was identified in the evaluation of specific TLR expression in each group, neither as per type of endometriosis.

**Figure 1:**
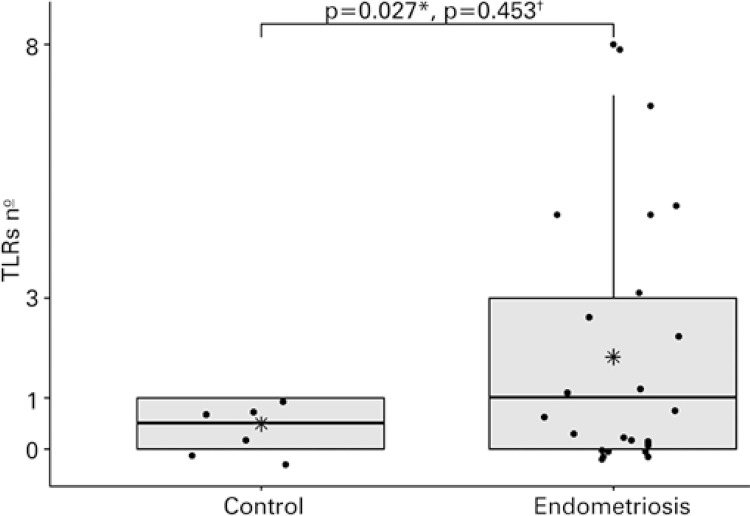
Number of Toll-like receptors expressed in regulatory T cells isolated from Endometriosis and Control Groups * Students *t*-test; ^†^ Wilcoxon rank-sum test. TLR: Toll-like receptors.

The comparison of the number of TLRs between Endometriosis and Control Groups should suggest the presence of endometriosis could be related to the expression of a greater number of receptors (Figure 1). Since the amount of TLRs expressed did not show a normal distribution or homogeneous variances, and considering the small sample size, Wilcoxon rank-sum test was used, but no statistically significant results were obseved (p=0.453). In the view of the lower efficiency of this method for small samples, we have also included the Student’s *t* test, which showed a statistically significant difference (p=0.027). No correlation was identified in the assessment of the number of TLRs expressed according to the age of endometriosis patients.

## DISCUSSION

This is the first study evaluating the expression of different TLRs in Treg cells isolated from peritoneal fluid from patients with and without endometriosis.

The results showed patients with endometriosis presented Treg cells expressing a larger number and variety of TLRs when compared to Tregs from patients in the Control Group. TLR1 and TLR2 in Tregs cells were expressed in both groups. All other expressed TLRs types were only found in Tregs from the Endometriosis Group, although only it was possible to identify the expression in approximately 33% (7 out of 23) of endometriosis patients. Quantitative evaluation of the total number of expressed receptors demonstrated that Endometriosis Group has a greater number of TLRs in relation to the control through parametric analysis used, due to its efficiency for small samples.^16^ Therefore, the expression of different receptors suggests a cumulative effect on immune response balance. In addition, TLR5 was found only in patients who expressed three or more distinct TLRs, corresponding to 6 out of 7 patients.

For more conclusive responses, more patients should be analyzed in this study, the small sample size is the main limitation of this study.

Treg cells are a subpopulation of T cells that maintain immunological self-tolerance and homeostasis, control the suppression of excessive immune response to the host^16^ and have been suggested to play a role in immune impairment observed in endometriosis disease.^7-10^ Toll-like receptors are transmembrane proteins located on the cell surface or on the endosome wall, responsible for inducing the expression of various host defense mechanisms.^15,17^Some studies suggested that microorganisms can be directly detected by Tregs through TLR^18,19^ that can modulate the suppressive function of Tregs,^19-24^ which could supposedly be enhanced or reversed by TLR agonists.^19^

The larger variety of TLRs types expressed by Tregs present in the peritoneal cavity of some patients with endometriosis could suggest the putative stimulation of the target cells by microorganisms. These could theoretically reach the peritoneal cavity by retrograde menstruation.^12^

Toll-like receptors agonists present in microorganisms can influence Treg functions.^19,24^ TLR2 and TLR8 agonists can inhibit the suppressive function of Tregs, whereas TLR4 and TLR5 agonists can have the opposite effect.^19^ Since microorganisms were not isolated, it was not possible to evaluate its direct association with Treg phenotype, but one can presume that at least for part of the women affected by endometriosis, the presence of microorganisms in the peritoneal cavity could modify, via Tregs, the balance of immune or inflammatory cell populations. The imbalance towards a more tolerogenic microenvironment would impair the normal elimination of endometrial cells carried to the peritoneal cavity.

## CONCLUSION

Although a group of endometriosis patients had peritoneal regulatory T cells expressing a larger number and variety of toll-like receptors compared to the corresponding type of the Control Group, statistical analysis was unable to achieve statistical significance. For these reasons, a study with larger cohort of patients is needed to prove our hypothesis and investigate phenotypic and functional changes in peritoneal cells in endometriosis.
