# Supplementary material for: Regulatory T cells isolated from endometriotic peritoneal fluid express a different number of Toll-like receptors
Source: Einstein (Sao Paulo). 2020 Mar 19;18:eAO5294. doi: 10.31744/einstein_journal/2020AO5294 (PMC7087457; doi:10.31744/einstein_journal/2020AO5294)
Supplement: Translation

# Translation: Células T reguladoras isoladas do fluido peritoneal de mulheres com endometriose expressam um número diferente de receptores do tipo Toll

**Authors:** Camila Hernandes, Bárbara Yasmin Gueuvoghlanian-Silva, Vitor Ulisses Monnaka, Natalia Mazini Ribeiro, Welbert de Oliveira Pereira, Sérgio Podgaec

**Affiliations:** 1 Brasil Hospital Israelita Albert Einstein, São Paulo, SP, Brasil.; 2 Brasil Instituto Israelita de Ensino e Pesquisa Albert Einstein, Hospital Israelita Albert Einstein, São Paulo, SP, Brasil.; 3 Brasil Faculdade Israelita de Ciências da Saúde Albert Einstein, Hospital Israelita Albert Einstein, São Paulo, SP, Brasil.

**Keywords:** Linfócitos T reguladores, Receptores toll-like, Sistema imunitário, Endometriose

## Abstract

**Objetivo:**

Analisar e comparar a expressão de receptores do tipo Toll por células T reguladoras presentes no líquido peritoneal de pacientes com endometriose.

**Métodos:**

Células T reguladoras foram isoladas do líquido peritoneal de mulheres com e sem endometriose, coletadas durante a cirurgia, e o RNAm foi extraído para análise da expressão de receptores do tipo Toll por reação em cadeia da polimerase com transcriptase reversa.

**Resultados:**

Pacientes com endometriose apresentaram células T reguladoras expressando maior número e variedade de Toll por células quando comparadas com T reguladoras de pacientes do Grupo Controle. Receptores do tipo Toll-1 e receptores do tipo Toll-2 foram expressos em ambos os grupos. Todos os outros tipos de receptores Toll foram encontrados expressos apenas em células T reguladoras do grupo com endometriose.

**Conclusão:**

Pacientes com endometriose apresentaram células T reguladoras peritoneais expressando vários tipos de receptores tipo Toll.

## INTRODUÇÃO

A patogênese da endometriose permanece incerta.^1,2^ A teoria mais aceita é a de Sampson;^3^ a qual propõe que, durante a menstruação, as células endometriais são transportadas pelas tubas uterinas por fluxo reverso até a cavidade peritoneal, onde aderem às superfícies da serosa. No entanto, embora a menstruação retrógrada ocorra em 90% das mulheres, apenas cerca de 10% são acometidas pela endometriose.^4,5^ Devido a essa discrepância, teorias complementares sugerem que a resposta imunológica de pacientes com endometriose fica alterada e não consegue eliminar os implantes endometriais ectópicos.^6-10^

Por exemplo, os linfócitos T reguladores (Treg) são uma subpopulação de linfócitos T especializados na regulação da resposta imune, que já foram implicados no surgimento da endometriose.^9^

Embora as células Treg estejam associados à produção de citocinas anti-inflamatórias, há evidências de que, sob condições inflamatórias, eles também expressam citocinas inflamatórias e, quando desregulados, contribuem para a manutenção da doença.^11^

Recentemente, uma nova teoria foi proposta, associando a presença de microrganismos ao desenvolvimento da endometriose.^11,12^ Essa teoria parte de estudos que indicam uma maior contaminação bacteriana no sangue menstrual, principalmente por *Escherichia coli*, e maiores níveis de endotoxina no fluido peritoneal de mulheres com endometriose, em comparação a pacientes sem a doença.^11,13^ Outro estudo mostrou que, em mulheres com endometriose, a presença de *Mycoplasma genitalium* é associada com a regulação negativa de genes relacionados à resposta inflamatória.^14^ A ideia é que, durante a menstruação retrógrada, tecido endometrial e microrganismos são carregados para a cavidade peritoneal,^12^ com posterior adesão, proliferação, diferenciação e invasão por essas células.^2^

Estudos sugerem que microrganismos podem ser diretamente detectados pelos linfócitos Treg por meio dos receptores do tipo Toll (TLRs), capazes de modular as funções supressoras dos Tregs, e essa função supressora dos linfócitos Treg pode ser potencializada ou revertida pelos TLRs.^15^

Considerando-se que mulheres com endometriose, de fato, apresentam mais microrganismos na cavidade peritoneal que mulheres saudáveis, e os microrganismos expressam e secretam agonistas de TLR, que poderiam afetar os linfócitos Treg, nossa hipótese é a de que há uma diferença entre a expressão de TLR em linfócitos Treg isolados de pacientes com endometriose em comparação aos controles.

## OBJETIVO

Analisar e comparar a expressão de receptores do tipo Toll por células T reguladoras isoladas de fluido peritoneal obtidos de pacientes com endometriose e de mulheres sem endometriose.

## MÉTODOS

### População do estudo

Este estudo de caso-controle foi realizado no Hospital Israelita Albert Einstein (HIAE), em São Paulo (SP), de 1º de janeiro a 1º de dezembro de 2018. O estudo foi aprovado pelo Comitê de Ética em Pesquisa do HIAE, número 563.939, CAAE: 25915014.2.0000.0071, e todas as pacientes assinaram um Termo de Consentimento Livre e Informado. As pacientes foram divididas em dois grupos com base nos achados cirúrgicos: com e sem endometriose. O diagnóstico de endometriose dependia da observação de lesões características durante a cirurgia e posterior confirmação por histologia. O Grupo Controle foi composto por mulheres sem qualquer evidência de endometriose, que tinham sido operadas por cistos benignos de ovário, miomas uterinos, laparoscopia diagnóstica ou laqueadura.

Foram incluídas 29 pacientes neste estudo: 23 com endometriose e 6 controles. Os critérios de inclusão foram mulheres com idade entre 18 e 40 anos com ciclos eumenorreicos, submetidas à cirurgia laparoscópica (para confirmar ou excluir endometriose). Os critérios de exclusão foram mulheres com doença autoimune, inflamatória e/ou neoplásica e/ou em terapia hormonal, incluindo análogos do hormônio de liberação de gonadotrofinas (GnRH), progestinas ou anticoncepcionais combinados, por 3 meses antes da cirurgia.

Para coleta de amostras, logo após a passagem do trocarte auxiliar na cirurgia laparoscópica, todo o fluido peritoneal depositado na escavação retouterina anterior e/ou posterior foi coletado com uma agulha laparoscópica.

As pacientes foram caracterizadas clinicamente pela presença de dispareunia profunda, dismenorreia, dor pélvica crônica, abortos, infertilidade, fase do ciclo menstrual, idade, etnia e índice de massa corporal (IMC). A dismenorreia foi medida por meio da Escala Visual Analógica (VAS), uma escala validada na qual uma linha de 10cm representa uma graduação entre “nenhuma dor” e “pior dor”.

### Isolamento e caracterização de linfócitos T reguladores provenientes do fluido peritoneal

O fluido peritoneal foi processado para que os linfócitos fossem isolados. Os eritrócitos foram lisados com tampão de lise em temperatura ambiente por 15 minutos. Em seguida, os linfócitos foram lavados com tampão fosfato-salino (PBS), contados e congelados em soro fetal bovino (SFB) (Gibco, USA) com dimetilsulfóxido (DMSO) a 10% (Sigma-Aldrich, USA) e criopreservados em nitrogênio líquido até o uso, quando foram descongelados sob agitação em banho-maria a 37°C. Os linfócitos foram lavados por centrifugação (250g, 8 minutos, 8°C) inicialmente com 0,01M PBS, pH 7,2, seguido por uma segunda lavagem em meio de cultura (R-10) (Invitrogen-Gibco, Gaithersburg, MD, USA). Depois, as células foram suspensas em meio R-10 para contagem e avaliação da viabilidade celular em um contador de células automático Countess™. O CD4^+^CD25^+^CD127^dim^ regulatory T Cell Isolation Kit II (MACS, MiltenyiBiotec, Auburn, CA, USA) foi usado para separar as células de acordo com as instruções do fabricante. Rapidamente, os linfócitos não CD4^+^ e CD127^high^ foram marcados com um coquetel de anticorpos conjugados com biotina (CD8, CD19, CD123 e CD127) e submetidos à separação magnética. Esta fração enriquecida de linfócitos T CD4^+^ foi marcada com anticorpos anti-CD25 e depois, novamente, submetida à separação magnética. Os Treg (CD4^+^CD25^+^CD127^dim^) foram eluídos como a fração de células selecionadas positivamente. As amostras foram centrifugadas, suspensas em 200μL de Trizol (reagente TRIzol^®^, Invitrogen) e congeladas a -80°C para extração total do ácido ribonucleico (RNA).

### Extração de RNA e teste de expressão do RNA mensageiro

O RNA total dos Tregs isolados foi extraído de acordo com um protocolo padrão de isolamento com Trizol e purificado (Rneasy Micro Kit™, Qiagen, Dusseldorf, Germany). A concentração de RNA foi determinada usando-se um espectrofotômetro NanoDrop^®^ ND-1000 (Nano-Drop Technologies, Wilmington, Australia). Todas as amostras foram armazenadas a -80°C.

Foi realizada síntese de ácido desoxirribonucleico complementar (cDNA) por transcrição reversa usando-se o *kit* específico ImProm-II™ Reverse Transcription System (Promega, Madison, WI, USA). As amostras foram incubadas com o volume específico de Random Primer a 70°C por 5 minutos e, depois, colocadas em gelo por 5 minutos. Depois, as amostras foram centrifugadas brevemente e deixadas em gelo até a conclusão do próximo passo. Uma mistura de reagentes contendo desoxinucleotídeo trifosfato (dNTP), MgCl_2_, transcriptase reversa ImProm-II (Promega) e tampão de reação ImProm-II™ 5x (Promega) foi preparada, adicionada ao RNA com Random Primer e incubada a 25°C por 5 minutos, 42°C por 60 minutos, seguida por 10 minutos a 70°C. Os cDNAs resultantes foram armazenados a -20°C. Todas as reações foram realizadas a uma concentração de RNA de 20ng/mL.

Uma pré-amplificação complementar de ácido desoxirribonucleico foi realizada utilizando-se uma mistura de reagentes composta pelos testes Taqman de expressão gênica (Applied Biosystems, Foster City, CA, USA) e tampão TE para completar o volume. Tubos com a mistura de reagentes, amostras de cDNA e o Master Mix Pre-Amp (Applied Biosystems) foram incubados a 95°C por 10 minutos e, depois, por dez ciclos de 95°C por 15 minutos, seguidos por 4 minutos a 60°C. Todas as amostras foram armazenadas a -20°C.

A expressão de RNA mensageiro foi determinada por reação em cadeia da polimerase com transcriptase reversa (RT-PCR) em um sistema de detecção de sequências ABI 7500 (Applied Biosystems). A reação em cadeia da polimerase foi realizada em tubos separados para cada reação, e cada amostra foi processada em duplicata. Foram usados testes TaqMan de expressão gênica (Applied Biosystems) para os genes-alvo TLR1 (Hs00413978_m1), TLR2 (Hs00610101_m1), TLR3 (Hs01551078_m1), TLR4 (Hs00152939_m1), TLR5 (Hs00152825_m1), TLR6 (Hs00271977_s1), TLR7 (Hs00152971_m1), TLR8 (Hs00152972_m1) e TLR9 (Hs00152973_m1).

### Análise estatística

As análises de dados foram realizadas no programa estatístico R. O nível de significância de 0,05 foi adotado para todas as comparações.

A distribuição de dados e variações de todas as variáveis contínuas foram analisadas pelos testes de Shapiro-Wilk e F, respectivamente. Considerando-se essas análises e o tamanho pequeno da amostra, foram usados testes não paramétricos. Para avaliar diferenças clínicas entre os grupos que poderiam influenciar nos resultados, comparou-se a presença de dispareunia profunda, dismenorreia, dor pélvica crônica, abortos, infertilidade, fase do ciclo menstrual, idade, etnia e IMC. Abortos, dispareunia profunda e dor pélvica crônica foram comparadas pelo teste exato de Fisher, considerando-se o pequeno tamanho da amostra e a tabela 2x2. Etnia, fase menstrual e infertilidade foram comparados pelo teste χ^2^. Idade, IMC e dismenorreia foram comparados pelo teste de soma de postos de Wilcoxon. Como não havia dados sobre etnia de quatro pacientes e sobre IMC de cinco pacientes, a análise foi realizada com os dados disponíveis.

A expressão de TLR em células Treg do fluido peritoneal dos Grupos de Endometriose e Controle foi analisada qualitativamente considerando-se o valor de limiar do ciclo (em inglês, *cycle threshold*, ou CT) de 32 para categorização da expressão dos receptores em uma variável binária qualitativa, “expresso” ou “não expresso”.

Em seguida, comparamos a expressão de TLRs específicos em cada grupo. Também avaliamos a expressão de TLR de acordo com o tipo de endometriose. Em ambos os casos, foi usado o teste exato de Fisher. O número de TLRs expressos em cada grupo foi comparado pelo teste de soma de postos de Wilcoxon. Considerando-se a menor eficiência deste método para amostras pequenas, incluímos também o teste *t* de Student. A correlação entre o número de TLRs expressos e a idade das pacientes com endometriose foi analisada pelo teste de Spearman.

## RESULTADOS

### Parâmetros clínicos

As características clínicas são apresentadas na tabela 1. Infertilidade foi reportada por 14 pacientes no Grupo de Endometriose e duas nos controles (p=0,019). A média para dismenorreia na EVA foi 8,37 no Grupo de Endometriose e 5,83 nos controles (p=0,027). Não houve diferença entre os grupos para os outros parâmetros clínicos.

**Tabela 1:** Características iniciais de pacientes com endometriose e controles

Parâmetros	Controle	Endometriose	Valor de p
Pacientes	6	23	
Idade	34,7	34,5	0,765
Fase mentrual			0,200
Menstrual	0	2	
Proliferativa	4	5	
Secretória	1	9	
Desconhecida	1	7	
Dismenorreia	5,83	8,39	0,027
Dor pélvica crônica	2	15	0,198
Dispareunia profunda	2	14	0,364
Infertilidade			0,019
Sim	2	14	
Não	4	3	
Desconhecida	0	6	
Abortos	1	4	1

Resultados expressos por n ou média.

Dismenorreia: média na Escala Visual Analógica. Abortos, dispareunia profunda e dor pélvica crônica foram comparados pelo teste exato de Fisher; fase menstrual e infertilidade, pelo teste χ^2^; idade e dismenorreia, pelo teste de soma dos postos de Wilcoxon.

### Expressão de receptores do tipo Toll

A expressão de TLRs nos linfócitos Treg obtidos do fluido peritoneal foi resumida na tabela 2. No Grupo Controle, três das seis mulheres não expressaram nenhum TLR em Tregs peritoneais, uma expressou apenas TLR1, e duas expressaram apenas TLR2. A expressão de TLR 3 a 9 por linfócitos Treg peritoneais foi negativa em todos os indivíduos do Grupo Controle.

**Tabela 2:** Expressão de receptores do tipo Toll por linfócitos T reguladores peritoneais de pacientes com endometriose e controles

TLR	Endometriose	Controle	Valor de p
TLR	12/23 (52,2)	3/6 (50,0)	1,000
TLR1	8/23 (34,8)	1/6 (16.7)	0,633
TLR2	12/23 (52,2)	2/6 (33.3)	0,651
TLR3	1/23 (4,3)	0/6 (0.0)	1,000
TLR4	5/23 (21,7)	0/6 (0.0)	0,553
TLR5	6/23 (26,1)	0/6 (0.0)	0,295
TLR6	2/23 (8,7)	0/6 (0.0)	1,000
TLR7	5/23 (21,7)	0/6 (0.0)	0,553
TLR8	3/23 (13,0)	0/6 (0.0)	1,000
TLR9	0/23 (0,0)	0/6 (0.0)	-

Resultados expressos pela (%) de mulheres dentro do grupo com e sem endometriose cujas células Treg apresentaram expressão de determinado tipo de TLR.

A expressão de um receptor Toll específico na população de linfócitos T reguladores peritoneais foi considerada positiva (+; variável binária, qualitativa), considerando-se o valor limiar do ciclo de 32. Receptores do tipo Toll comparados pelo teste exato de Fisher. TLR: receptores do tipo Toll.

No Grupo de Endometriose, sete pacientes apresentaram Tregs peritoneais com expressão de três ou mais TLRs, sendo TLR5 o mais frequente (em seis de sete pacientes). Quatro pacientes expressaram apenas TLR2, uma paciente expressou tanto TLR1 quanto TLR2, e em 11 pacientes, os Tregs peritoneais não expressaram TLR.

Exceto pelo TLR9, foi observada expressão de todos os TLRs (sozinhos ou coexpressos) em Tregs de pacientes com endometriose.

Não foi identificada associação na avaliação da expressão de TLR específicos em cada grupo, nem de acordo com o tipo de endometriose.

A comparação do número de TLR entre os Grupos de Endometriose e Controle deveria sugerir que a presença da endometriose pode estar relacionada à expressão de um maior número de receptores (Figura 1). Como o número de TLR expressos não mostrou distribuição normal nem variâncias homogêneas e considerando-se o tamanho pequeno da amostra, o teste de soma de pontos de Wilcoxon foi usado, mas não foram observados resultados estatisticamente significativos (p=0,453). Tendo em vista a eficiência mais baixa desse método com amostras menores, incluímos também o teste *t* de Student, que mostrou diferença estatisticamente significante (p=0,027). Nenhuma correlação foi identificada (dados não mostrados) na avaliação do número de TLRs expressos, de acordo com a idade das pacientes com endometriose.

**Figura 1:** Número de receptores do tipo Toll expressos em células T reguladoras isoladas dos Grupos Endometriose e Controle * Teste *t* de Student; ^†^ teste de soma de postos de Wilcoxon. TLR: receptores do tipo Toll.

## DISCUSSÃO

Este é o primeiro estudo que avaliou a expressão de diferentes TLRs em células Treg isoladas do fluido peritoneal de pacientes com e sem endometriose.

Os resultados mostram que pacientes com endometriose apresentaram células Treg com expressão de maior número e variedade de TLRs em comparação aos linfócitos Treg de pacientes do Grupo Controle. TLR1 e TLR2 foram expressos pelos linfócitos Treg em ambos os grupos. Todos os outros tipos de TLRs foram encontrados somente em Tregs do Grupo de Endometriose, embora só tenha sido possível identificar essa expressão em cerca de 33% (7 de 23) das pacientes com endometriose. A avaliação quantitativa do número total de receptores expressos demonstrou que o Grupo de Endometriose apresenta um número maior de TLRs em relação aos controles, usando-se análise paramétrica, devido à eficiência desta para amostras pequenas.^16^ Assim, a expressão de diferentes receptores sugere um efeito cumulativo sobre o balanço da resposta imune. Além disso, o TLR5 foi encontrado apenas em pacientes que expressavam três ou mais TLRs diferentes, correspondendo a seis de sete pacientes.

Para respostas mais conclusivas, este estudo deveria ter analisado mais pacientes, e o tamanho pequeno da amostra é sua principal limitação.

As células Treg são uma subpopulação de células T que mantêm a autotolerância imunológica e a homeostase, controlam a supressão da resposta imune excessiva ao hospedeiro,^16^ e supostamente influenciam no comprometimento imunológico observado na endometriose.^7-10^ Os TLRs são proteínas transmembrana localizadas na superfície das células ou na parede do endossomo, responsáveis por induzir a expressão de vários mecanismos de defesa do hospedeiro.^15,17^ Alguns estudos sugeriram que microrganismos podem ser diretamente detectados por linfócitos Treg por meio dos TLRs,^18,19^ receptores capazes de modular a função supressora dos Tregs,^19-24^ e que essa função supressora das células Treg pode, supostamente, ser potencializada ou revertida pelos agonistas de TLR.^19^

A maior variedade de tipos de TLRs expressos por Tregs presentes na cavidade peritoneal de pacientes com endometriose poderia sugerir uma suposta estimulação das células-alvo por microrganismos. Estes, teoricamente, chegariam à cavidade peritoneal pela menstruação retrógrada.^12^

Os agonistas de receptores Toll presentes nos microrganismos podem influenciar nas funções dos linfócitos Treg.^19,24^ Os agonistas de TLR2 e TLR8 podem inibir a função supressora dos linfócitos Treg, enquanto os agonistas de TLR4 e TLR5 podem ter o efeito oposto.^19^ Como os microrganismos não foram isolados, não foi possível avaliar sua associação direta com o fenótipo dos Treg, mas pode-se supor que, pelo menos para algumas das mulheres acometidas pela endometriose, a presença de microrganismos na cavidade peritoneal poderia modificar, por meio dos Tregs, o balanço entre as populações de células imunes ou inflamatórias. O desequilíbrio em direção a um microambiente mais tolerogênico prejudicaria a eliminação normal das células endometriais carregadas para a cavidade peritoneal.

## CONCLUSÃO

Embora um grupo de pacientes com endometriose tenha apresentado células T reguladoras peritoneais com expressão de receptores do tipo Toll em maior número e variedade em comparação ao tipo correspondente no Grupo Controle, a análise não foi capaz de alcançar significância estatística. Assim, é necessário um estudo com coortes maiores de pacientes para comprovar nossa hipótese e investigar alterações fenotípicas e funcionais em células peritoneais na endometriose.
